# Rare Earth Doped Glasses/Ceramics: Synthesis, Structure, Properties and Their Optical Applications

**DOI:** 10.3390/ma15228099

**Published:** 2022-11-16

**Authors:** Wojciech A. Pisarski

**Affiliations:** Institute of Chemistry, University of Silesia, Szkolna 9 Street, 40-007 Katowice, Poland; wojciech.pisarski@us.edu.pl

Glasses, glass-ceramics and ceramics belong to three important classes of engineering materials, which are useful in numerous multifunctional and industrial applications [1]. Ceramic materials have attracted a lot of attention due to their advantages. They are ideally suitable to incorporate rare-Earth ions. Thus, ceramic materials with rare-Earth ions are excellent candidates for UV, visible or IR-persistent phosphors [2] and solid-state laser applications [3]. Inorganic glasses belong to the unique amorphous solid-state materials, which are usually thermally stable and formed over a wide region of glass-former concentrations. Since the first demonstration of laser action of Nd^3+^ ions in barium crown glass by Snitzer in 1961 [4], a great number of published works has been carried out on rare-Earth-doped glasses for near-IR lasers [5], broadband optical amplifiers [6], up-conversion luminescence temperature sensors [7] and solid-state-lighting (SSL) applications [8]. Further, several precursor glasses were applied to fabricate optical fibers emitting IR radiation [9]. Transparent glass-ceramics [10], referred to as TGC, are known in the literature data as a new composite material, with their general properties between crystals and glasses. Thermal treatment or laser irradiation introduces structural transformation from precursor glass to transparent glass-ceramics with oxide or fluoride crystals, usually on the nanometric scale. Rare-Earth ions, as optically active ions, are usually incorporated into the crystalline phases. Recently, glass-ceramics have been examined for optical amplifiers, photovoltaic devices, color displays, optical limiters and random lasers [11]. These phenomena are interesting and important from scientific and technological points of view. Systematic studies of radiative and non-radiative relaxation processes and their mechanisms between rare-Earth ions are necessary for knowledge about optical glasses, glass-ceramics and ceramics. These aspects are presented and discussed in this Special Issue, belonging to the section “Advanced and Functional Ceramics and Glasses”.

The aim of this Special Issue is to present novel results for luminescent glasses, glass-ceramics and ceramic materials, which offer important contributions to the development of scientific research in the field of glass/ceramic science and technology, modern photonics and applied spectroscopy. The themed collection includes 11 articles published in the last two years, concerning rare-Earth-doped glasses/ceramics: from synthesis, structure and properties to their potential optical applications.

Synthesis and characterization of germanate ceramics Li_2_MgGeO_4_:Ho^3+^ are reported by Bednarska-Adam et al. [12]. The studied ceramic system belongs to the monoclinic Li_2_MgGeO_4_, which was verified using X-ray diffraction measurements. Spectroscopic analysis revealed the presence of broad blue luminescence, assigned to ceramic matrix and narrow emission bands characteristic for the 4f-4f transitions of holmium ions. The broad blue emission band is associated with the presence of magnesium in ceramic matrix and the occurrence of defects and oxygen vacancies assigned to F-type centers [13].

The effects of the crystalline size of nanophosphors Y_3_Al_1.99_Cr_0.01_Ga_3_O_12_ (YAGG:Cr^3+^) on their optical properties were studied by Boiko et al. [14]. Ceramic phosphors were synthesized on the nanometric scale by the Pechini method and then annealed at various temperatures (900, 1100 and 1300 °C). Formation of a garnet-type structure was well observed for the synthesis of nanophosphors YAGG:Cr^3+^ at T = 900 °C [15]. The authors concluded that the higher annealing temperature (>1100 °C) did not improve the optical properties of the studied ceramic phosphors. The nanophosphors YAGG:Cr^3+^ annealed at T = 1100 °C show an acceptable degree of particle agglomeration and may be suitable for practical applications as starting materials for the production of high-quality optical ceramics and luminescent markers for imaging in the first biological window.

The performances of antimony–germanate–silicate glass-ceramics containing Eu^3+^ were elaborated by Żmojda et al. [16]. In particular, the mechanisms of crystallization of EuPO_4_ nanocrystals located in antimony–germanate–silicate glasses and their optical properties were investigated in detail. Based on measurements of emission spectra and their decays, the optimal molar concentrations of P_2_O_5_ and optical dopants (Eu^3+^) in TGC systems were determined. The optimal way to fabricate the antimony–germanate–silicate glass-ceramics in the form of optical fibers is still the appropriate compromise between their luminescent properties and fiber-drawing ability.

Kowalska et al. [17] report near-IR luminescence properties of selected rare-Earth ions (Er^3+^, Pr^3+^, Ho^3+^, Tm^3+^) in titanate–germanate glasses under excitation of Yb^3+^. The resonant Yb^3+^ → Pr^3+^ and Yb^3+^ → Er^3+^ and non-resonant Yb^3+^ → Tm^3+^ and Yb^3+^ → Ho^3+^ energy transfer in co-doped titanate–germanate glass is quite well observed. The introduction of TiO_2_ to germanate glass enhances the near-IR emission bands located at 1.3 µm (Pr^3+^: ^1^G_4_ → ^3^H_5_), 1.5 µm (Er^3+^: ^4^I_13/2_ → ^4^I_15/2_), 1.8 µm (Tm^3+^: ^3^F_4_ → ^3^H_6_) and 2 µm (Ho^3+^: ^5^I_7_ → ^7^I_8_). Systematic studies revealed that the lifetimes of Yb^3+^ ions are reduced with increasing TiO_2_ content, whereas the energy transfer efficiencies are changed completely different, depending on a pair of Yb^3+^/Ln^3+^ (Ln = Pr, Er, Tm, Ho) in titanate–germanate glass.

The next paper published in this Special Issue is concentrated on inorganic glasses singly doped with Pr^3+^ ions [18]. Spectroscopic properties of Pr^3+^ ions in borate-based glass with Ga_2_O_3_ and BaO, lead–phosphate glass with Ga_2_O_3_, gallo-germanate glass modified by BaO/BaF_2_ and multicomponent fluoride glass based on InF_3_ were compared. Visible emission of Pr^3+^ is modulated from red/orange for borate glass and lead–phosphate glass with Ga_2_O_3_ via yellowish orange for gallo-germanate glass with BaO/BaF_2_ to nearly white light for fluoride glass based on InF_3_. The positions and spectral linewidths for near-IR emission bands at the optical telecommunication window corresponding to the ^1^G_4_ → ^3^H_5_, ^1^D_2_ → ^1^G_4_ and ^3^H_4_ → ^3^F_3_,^3^F_4_ transitions of Pr^3+^ ions depend strongly on glass matrices and excitation wavelengths. The authors concluded that low-phonon InF_3_-based fluoride glass and gallo-germanate glass with BaO/BaF_2_ are excellent candidates for broadband near-IR optical amplifiers operating at E-, S-, C- and L-bands [19].

The influence of TeO_2_/GeO_2_ molar ratios on spectroscopic properties of Eu^3+^-doped oxide glasses is reported by Leśniak et al. [20]. Glasses were investigated to explore their potential application as an efficient host material for rare-Earth doping and optical fiber drawing and characterized using different experimental techniques, i.e., DSC, Raman, MIR, refractive index, PLE, PL spectra and time-resolved spectral measurements. Several spectroscopic parameters, among others, the fluorescence intensity ratio R/O and the measured lifetime of Eu^3+^, were determined. Based on luminescence measurements, glass with the highest content of GeO_2_ was selected for optical preform and fiber fabrication.

Another article from this Issue deals with rare-Earth (Eu^3+^) and transition metal (Cr^3+^) in glasses containing extremely different glass-formers B_2_O_3_ and GeO_2_ [21]. The Cr^3+^ ions are located at octahedral sites, which was evidenced by EPR spectroscopy. Independently of the molar ratio of glass-formers GeO_2_:B_2_O_3_, red emission of Cr^3+^ was successfully observed. Intensities of emission bands originating to transitions of Eu^3+^ and fluorescence intensity ratio R/O are reduced with increasing B_2_O_3_ content. On the other hand, the increase in B_2_O_3_ concentration results in an enhancement in the ^5^D_0_ lifetime of Eu^3+^. The results clearly indicate that spectroscopic properties of transition metals and rare-Earth ions should also be controlled by the choice of glass-network-formers.

Structural and photoluminescence investigations of silicate glass-ceramics with CaF_2_ nanocrystals co-doped with Tb^3+^/Eu^3+^ ions are described by Pawlik et al. [22]. The series of glass-ceramics with CaF_2_ nanocrystals were fabricated using the sol–gel method, which is a suitable alternative for photonic materials [23]. The presence of an energy transfer process between Tb^3+^ and Eu^3+^ was evidenced by the excitation/emission spectra measurements. In particular, the emission decay curve analysis confirmed partial segregation of Tb^3+^ and Eu^3+^ ions inside CaF_2_ nanocrystals formed during a controlled heat treatment process.

Spectroscopic properties of rare-Earth ions have also been examined for glasses with the general formula PbO-Ga_2_O_3_-Me_x_O_y_-Ln_2_O_3_ (Me = Ge, Si, P, B and Ln = Eu, Er), belonging to a wide family of heavy metal glasses [24,25,26]. This topic was addressed by Pisarska et al. [27]. The experimental results are presented and discussed in two parts of the published paper. The first part consists of phonon sideband analysis from the excitation spectra of Eu^3+^. The second part is concerned with the near-IR emission of Er^3+^ ions in heavy-metal-oxide glasses. Correlation between the measured lifetimes of rare-Earth ions, the energy gaps between interacting levels and the phonon energies of these glass systems are well evidenced.

The next paper, published by Górny et al. [28], summarizes comparative analysis for lead borate glasses and glass-ceramics singly doped with Dy^3+^ ions. In particular, the Commission Internationale de I’Eclairage (CIE) chromaticity coordinates (x, y) were calculated for glasses and glass-ceramics in relation to potential applications for white light-emitting diodes (W-LEDs). It was well demonstrated that spectroscopic properties and chromaticity coordinates of lead borate systems can be effectively controlled by modification of the chemical composition of glass hosts and changing the excitation wavelengths.

Kuwik et al. [29] describe the influence of oxide glass-modifiers on the structural and spectroscopic properties of phosphate glasses doped with rare-Earth ions. The authors used the modifier MO (M = Ca, Sr, Ba) to improve spectroscopic properties of Eu^3+^ and Er^3+^ ions in phosphate glass. Independently of the kind of glass modifier, the intense red (611 nm) and near-IR (1500 nm) emissions were observed for Eu^3+^ and Er^3+^. Fluorescence intensity ratio R/O due to ^5^D_0_ → ^7^F_2_ (red) and ^5^D_0_ → ^7^F_1_ (orange) transition of Eu^3+^ depends significantly on the modifier MO (M = Ca, Sr, Ba). The lifetimes ^5^D_0_ (Eu^3+^) and ^4^I_13/2_ (Er^3+^) increase with increasing ionic radius of alkaline earth ion CaO < SrO < BaO. The results suggest the applicability of phosphate glasses with oxide modifiers (CaO, SrO, BaO) as potential red (Eu^3+^) and near-IR (Er^3+^) luminescent materials in photonic devices.

## References

[1] Baino F., Tomalino M., Tulyaganov D. (2021). Ceramics, Glass and Glass-Ceramics: From Early Manufacturing Steps towards Modern Frontiers.

[2] Poelman D., Van der Heggen D., Du J., Cosaert E., Smet P.F. (2020). Persistent phosphors for the future: Fit for the right application. J. Appl. Phys..

[3] Tian F., Ikesue A., Li J. (2022). Progress and perspectives on composite laser ceramics: A review. J. Eur. Ceram. Soc..

[4] Snitzer E. (1961). Optical maser action of Nd^3+^ in a barium crown glass. Phys. Rev. Lett..

[5] Manasa P., Srihari T., Basavapoornima C., Joshi A.S., Jayasankar C.K. (2019). Spectroscopic investigations of Nd^3+^ ions in niobium phosphate glasses for laser applications. J. Lumin..

[6] Lin H., Jiang S., Wu J., Song F., Peyghambarian N., Pun E.Y.B. (2003). Er^3+^ doped Na_2_O–Nb_2_O_5_–TeO_2_ glasses for optical waveguide laser and amplifier. J. Phys. D Appl. Phys..

[7] Pisarski W.A., Pisarska J., Lisiecki R., Ryba-Romanowski W. (2016). Er^3+^/Yb^3+^ co-doped lead germanate glasses for up-conversion luminescence temperature sensors. Sens. Actuators A.

[8] Erol E., Vahedigharehchopogh N., Kıbrıslı O., Ersundu M.C., Ersundu A.E. (2021). Recent progress in lanthanide-doped luminescent glasses for solid-state lighting applications—A review. J. Phys. Condens. Matter.

[9] Ballato J., Ebendorff-Heidepriem H., Zhao J., Petit L., Troles J. (2017). Glass and process development for the next generation of optical fibers: A review. Fibers.

[10] Singh S.P., Sontakke A.D. (2021). Transparent glass ceramics. Crystals.

[11] de Araújo C.B., Kassab L.R.P., da Silva D.M. (2022). Optical properties of glasses and glass-ceramics for optical amplifiers, photovoltaic devices, color displays, optical limiters, and Random Lasers. Opt. Mater..

[12] Bednarska-Adam N., Kuwik M., Pietrasik E., Pisarski W.A., Goryczka T., Macalik B., Pisarska J. (2022). Synthesis and characterization of Li_2_MgGeO_4_:Ho^3+^. Materials.

[13] Martinez-Boubeta C., Martinez A., Hernandez S., Pellegrino P., Antony A., Bertomeu J., Balcells L., Konstantinovic Z., Martinez B. (2011). Blue luminescence at room temperature in defective MgO films. Solid State Commun..

[14] Boiko V., Dai Z., Chaika M., Grzeszkiewicz K., Li J., Strek W., Hreniak D. (2022). Size-dependent persistent luminescence of YAGG:Cr^3+^ nanophosphors. Materials.

[15] Boiko V., Dai Z., Markowska M., Leonelli C., Mortalò C., Armetta F., Ursi F., Nasillo G., Saladino M.L., Hreniak D. (2021). Particle size-related limitations of persistent phosphors based on the doped Y_3_Al_2_Ga_3_O_12_ system. Sci. Rep..

[16] Golonko P., Sadowska K., Ragiń T., Kochanowicz M., Miluski P., Dorosz J., Kuwik M., Pisarski W., Pisarska J., Leśniak M. (2022). Crystallization mechanism and optical properties of antimony-germanate-silicate glass-ceramic doped with europium ions. Materials.

[17] Kowalska K., Kuwik M., Pisarska J., Pisarski W.A. (2022). Near-IR luminescence of rare-earth ions (Er^3+^, Pr^3+^, Ho^3+^, Tm^3+^) in titanate-germanate glasses under excitation of Yb^3+^. Materials.

[18] Pisarska J., Kuwik M., Pisarski W.A. (2022). Spectroscopic properties of inorganic glasses doped with Pr^3+^: A comparative study. Materials.

[19] Liu X., Chen B.J., Pun E.Y.B., Lin H. (2012). Ultra-broadband near-infrared emission in praseodymium ion doped germanium tellurite glasses for optical fiber amplifier operating at E-, S-, C-, and L-band. J. Appl. Phys..

[20] Leśniak M., Zeid J., Starzyk B., Kochanowicz M., Kuwik M., Żmojda J., Miluski P., Baranowska A., Dorosz J., Pisarski W. (2022). Investigation of the TeO_2_/GeO_2_ ratio on the spectroscopic properties of Eu^3+^-doped oxide glasses for optical fiber application. Materials.

[21] Kowalska K., Kuwik M., Polak J., Pisarska J., Pisarski W.A. (2021). Transition metals (Cr^3+^) and lanthanides (Eu^3+^) in inorganic glasses with extremely different glass-formers B_2_O_3_ and GeO_2_. Materials.

[22] Pawlik N., Szpikowska-Sroka B., Goryczka T., Pisarska J., Pisarski W.A. (2021). Structural and photoluminescence investigations of Tb^3+^/Eu^3+^ co-doped silicate sol-gel glass-ceramics containing CaF_2_ nanocrystals. Materials.

[23] Gorni G., Velázquez J.J., Mosa J., Balda R., Fernández J., Durán A., Castro Y. (2018). Transparent glass-ceramics produced by sol-gel: A suitable alternative for photonic materials. Materials.

[24] Yamauchi H., Ohishi Y. (2005). Spectroscopic properties of Er^3+^-doped PbO–Ga_2_O_3_–GeO_2_ glass for optical amplifiers. Opt. Mater..

[25] Pisarski W.A., Pisarska J., Zur L., Goryczka T. (2013). Structural and optical aspects for Eu^3+^ and Dy^3+^ ions in heavy metal glasses based on PbO–Ga_2_O_3_–XO_2_ (X = Te, Ge, Si). Opt. Mater..

[26] Pisarski W.A., Pisarska J., Lisiecki R., Ryba-Romanowski W. (2016). Sensitive optical temperature sensor based on up-conversion luminescence spectra of Er^3+^ ions in PbO-Ga_2_O_3_-XO_2_ (X = Ge, Si) glasses. Opt. Mater..

[27] Pisarska J., Pisarski W.A., Lisiecki R., Ryba-Romanowski W. (2021). Phonon sideband analysis and near-infrared emission in heavy metal oxide glasses. Materials.

[28] Górny A., Kuwik M., Pisarski W.A., Pisarska J. (2020). Lead borate glasses and glass-ceramics singly doped with Dy^3+^ for white LEDs. Materials.

[29] Kuwik M., Pisarska J., Pisarski W.A. (2020). Influence of oxide glass modifiers on the structural and spectroscopic properties of phosphate glasses for visible and near-infrared photonic applications. Materials.

